# A qualitative study of factors influencing different generations of Newfoundland and Saskatchewan trained physicians to leave a work location

**DOI:** 10.1186/1478-4491-10-18

**Published:** 2012-07-25

**Authors:** Maria Mathews, Maureen Seguin, Nurun Chowdhury, Robert T Card

**Affiliations:** 1 Division of Community Health & Humanities, Health Sciences Centre, Memorial University of Newfoundland, Rm 2837, St. John’s, NL, A1B 3 V6, Canada; 2 Division of Hematology, Department of Medicine, Royal University Hospital, Room 2610, 103 Hospital Drive, Saskatoon, SK, S7N 0 W8, Canada; 3 Division of Community Health & Humanities, Health Sciences Centre, Memorial University of Newfoundland, Rm 2847a, St. John’s, NL, A1B 3 V6, Canada; 4 Division of Hematology, Department of Medicine, Royal University Hospital, Room 2610, 103 Hospital Drive, Saskatoon, SK, S7N 0 W8, Canada

**Keywords:** Generations, Physician supply, Retention, Turnover, Qualitative

## Abstract

**Background:**

Some studies have suggested that young physicians may have different expectations and practice behaviours than their older generational counterparts, including their reasons for wanting to remain or leave a community. This study examined the factors associated with a physician’s decision to leave a work location. We compared different generations of physicians to assess whether these factors have changed over generations.

**Methods:**

We conducted semi-structured, qualitative interviews with 48 physicians who graduated from two Canadian medical schools. We asked each physician about the number and nature of work location changes and the factors related to their decisions to leave each location. Interview transcripts and notes were analysed using a thematic analysis approach.

**Results:**

Dissatisfaction with the working environment was the most frequently cited reason for leaving a location for physicians of all generations. Elements which contributed to the quality of the work environment included the collaborative nature of the practice, the relationship with administrators, and access to resources and personnel. For younger physicians, the work environment had to meet their personal expectations for work-life balance. While remuneration level was given by some physicians as the key reason for leaving a location, for others it was the “last straw” if the work environment was poor. A small number of older generation physicians moved in response to political events and/or policies

**Conclusions:**

We documented generational differences in physicians’ reasons for choosing a work location. We found that a poor work environment was universally the most important reason why a physician chose to leave a location. A few physicians who were unsatisfied with their work location identified level of remuneration as an additional reason for leaving. Some older generation physicians cited political climate as a reason for leaving a work location. While economic factors have largely been the focus of recruitment and retention initiatives, our findings highlight the importance of the work environment and organizational culture on the retention of physicians of all generations.

## 

Despite increases in the number of physicians trained, provinces such as Saskatchewan and Newfoundland and Labrador (hereafter Newfoundland) continue to lose physicians to other jurisdictions. Between 2004 and 2008, the average annual net loss was 26.6 physicians for Newfoundland and 35.2 physicians for Saskatchewan [1]. A recent survey of Canadian physicians found that 0.6% of physicians moved to another country and 1.4% relocated from another Canadian province or territory in the preceding two years [2]. In Canada, various reports have suggested that the turnover rate in rural communities may be much higher and that between 18% and 30% of rural physicians leave their jobs each year [3,4]. A survey of British Columbian physicians found that 35 of 195 (18%) rural/remote physicians indicated they were planning to relocate to an urban setting [4]. In Saskatchewan, 52% of rural physicians left their communities over a five-year period, from fiscal 1992/1993 to 1996/1997 [5]. When surveyed about their intentions to remain in their community, two-thirds of the physicians in rural Saskatchewan reported that they intended to remain in their community in the next three years [5]. However, only 40% said they would remain in practice in rural Saskatchewan in the next five years. Studies conducted in the United States of America describe similar retention rates [6,7]. A survey of rural United States physicians reported that 27.1% intended to leave their practices in the next two years [6].

High physician turnover in Saskatchewan and Newfoundland contributes to the ongoing shortage of physicians, particularly in rural communities and in some specialties. A recent survey of Canadian physicians reported that nationally, 2.3% of physicians planned to move to another country and 3.6% planned to relocate to another Canadian province or territory in the next two years [2]. In contrast, 5.2% and 18.4% of Newfoundland physicians and 3.0% and 12.6% of Saskatchewan physicians planned to move to another country or to relocate to another Canadian province or territory in the next two years, respectively. Using data from the Scott’s Medical Data Base, the Canadian Institutes for Health Information [8] reported that 6% of physicians in Canada move from one community to another each year. Moreover, over a 10 year period, 25% of physicians moved from one community to another. The vast majority of these moves are within the same province.

Canadian studies have consistently reported that the distance away from friends and family as well as limited educational opportunities for children were common reasons for leaving rural practice [4,5,9,10]. Reasonable on-call schedules, an available locum program, satisfactory fees schedules, and access to specialists and referral networks have been cited by rural physicians as key to retaining rural physicians [11-15]. For example, among rural physicians in Saskatchewan, the limited ability to take vacation time as well as long work hours were the two greatest sources of dissatisfaction [5]. Among rural physicians from British Columbia, 35 of 195 (18%) were planning to move to an urban setting; among these physicians, the heavy on-call schedule was the most important factor in their decision to leave [4].

Most studies examining physician turnover and their reasons for leaving a community almost exclusively focus on rural physicians, providing little information on urban physicians and/or specialists. Moreover, these studies often focus on physicians who remain in a community and employ “intention to leave” questions [3-5,16] partly because of the difficulties in locating physicians who have left. Intention to leave questions may overestimate the number of physicians who leave a community and skew the actual reasons cited for turnover while studies on rural physicians may over-represent international medical graduates, who are more likely to leave a community and often have different reasons for initially moving to a rural location than their domestically-trained counterparts [17,18]. Lastly, studies focusing on physicians who remain in a location may not distinguish between the factors that promote retention versus those that lead to turnover.

In addition, some studies have suggested that young physicians may have different expectations and practice behaviours than their older generational counterparts, including their reasons for wanting to remain in or leave a community [16,19]. Understanding differences between generations and the expectations of younger, more recently graduated physicians is essential in the development of effective recruitment and retention polices. It is particularly important for jurisdictions like Newfoundland and Saskatchewan which have recently increased the number of students admitted to medical school as a means of alleviating projected physician shortages.

The purpose of this study is to examine the factors associated with a physician’s decision to leave a work location. We compared different generations of physicians (cohorts who graduated from medical school in the 1960s, 1970s, 1980s, and 1990s) to assess whether these factors have changed over generations. Understanding the reasons why physicians leave a work location will help identify public policy initiatives to address high physician turnover.

### Methods

We conducted qualitative interviews with different generations of physicians who graduated from the undergraduate medical programs at either Memorial University of Newfoundland or University of Saskatchewan. We defined generations on the basis of year of graduation. Early-career physicians graduated between 1995 and 1999; mid-career physicians graduated between 1985 and 1989; late-career physicians graduated between 1975 and 1979, and end-career physicians graduated between 1965 and 1969.

For each generation and each university, we interviewed two to five physicians who were working in the province where they completed their undergraduate medical school training (e.g., Memorial University graduates in Newfoundland and University of Saskatchewan graduates in Saskatchewan) and three to five physicians who were working outside the province where they trained. End-career physicians were interviewed only from University of Saskatchewan since Memorial University’s first medical class graduated in 1973 and were in the late-career category.

We randomly drew the names of physicians from the database of graduates that we had used in previous studies [20,21]. Recruitment was guided by saturation (i.e., when no new themes emerged from the data) as well as representation from strata (school/generation/location) of our recruitment framework. To be included in the study, physicians had to be living in Canada or the United States (to limit long-distance costs), be in active clinical practice or have retired within three years before the interview. We excluded physicians who were sponsored by foreign governments to study medicine.

Research assistants contacted and recruited physicians and conducted semi-structured interviews in English between July 2007 and December 2008. Interviews took place in person or by telephone and lasted between 10 and 45 minutes. All but two interviews were audio-recorded and transcribed verbatim. In these two interviews, the research assistant took detailed notes of the conversation.

In the interview, we asked each physician about the number and nature of work location changes over the course of their career and the factors related to their decision to choose or leave each location (i.e., a specific community). For each work location, we clarified which were chosen as part of post-graduate residency training and which were selected once the physician had completed training. For physicians who graduated before 1994, we clarified between jobs following the initial general internship training (as a general practitioner) and those after additional post-graduate training, if applicable. In our analysis, we focused on “permanent” job locations once post-graduate training was complete. We excluded locums and fellowships, but included work locations as a general practitioner if the physician returned for more post-graduate training afterwards. For the one participant who served in the military, we excluded the locations related to his military service. In this paper, we focus on the factors related to the decision related to leaving a work location

Probes for questions relating to factors for choosing or leaving a work location were based on the framework developed by Barer et al. [11] which suggests six groups of factors that underlie practice location decisions: 1) personal background factors, 2) professional education factors, 3) professional practice factors, 4) personal/family factors, 5) community factors, and 6) economic factors. Personal background includes gender, rural background (whether a physician or physician’s spouse grew up in a rural community), age, etc. Professional education factors relate to the physician’s training. Professional practice factors describe the practice environment. It includes, for example, availability of professional support and backup, the availability of a community hospital or medical centre, continuing education opportunities, practice group size, etc. Personal/family factors include the spouse’s preferences, suitability of professional/social peer group, educational and extra-curricular opportunities for children, proximity to family and friends, etc. Frequently studied community factors include climate, recreational and cultural opportunities, and socio-economic status of the community. Economic factors include gross income opportunities, practice costs, financial risk, and employment opportunities for the spouse.

Using a thematic analysis approach, two members of the research team independently read six transcripts in order to identify key words and themes [22]. Through this process of exploration, we developed a coding template that incorporated and built upon the framework of Barer et al. [11]. We compared the coding of an initial set of six interviews (three from each province) to ensure consistency of coding and to revise the coding template where necessary. Transcripts were then coded using NVivo software. In this article, we focus on the reasons for leaving a work location. While we were primarily interested in differences between generations, we also looked at similarities and differences between graduates from each university, location, gender and specialty (family physicians/general practitioners versus specialists).

The study received ethics approval from Memorial University and the University of Saskatchewan. Each physician consented to the interview and the use of their quotations. To protect the identities of physicians who participated in the study, data which could be used to identify individual physicians have been edited in the quotations.

### Results

We conducted interviews with 48 physicians whose characteristics are presented in Table 1. Almost three quarters of the physicians in our study worked in one or two locations. Older generation physicians had worked in more locations than early career physicians. Professional practice, economic and other factors influenced physicians to leave a work location.

**Table 1:** Characteristics of study participants by generation

	**Generation**				**Total no. (%)**
**Characteristics**	**End career no. (%)**	**Late career no. (%)**	**Mid-career no. (%)**	**Early career no. (%)**	
**Medical school**					
Saskatchewan	9 (100)	6 (50.0)	6 (40)	5 (41.7)	26 (54.2)
Memorial	n/a	6 (50.0)	9 (60)	7 (58.3)	22 (45.8)
**Gender**					
Male	9 (100)	5 (41.7)	11 (73.3)	7 (58.3)	32 (66.7)
Female	0	7 (58.3)	4 (26.7)	5 (41.7)	16 (33.3)
**In “home” province**					
Yes	4 (44.4)	6 (50.0)	7 (46.7)	6 (50.0)	23 (47.9)
No	5 (55.6)	6 (50.0)	8 (53.3)	6 (50.0)	25 (52.1)
**Specialty**					
Family/General Practitioner	5 (55.6)	5 (41.7)	3 (20.0)	2 (16.7)	15 (32.3)
Specialist	4 (44.4)	7 (58.3)	12 (80.0)	10 (83.3)	33 (68.7)
**Number of locations**					
1	3 (33.3)	3 (25.0)	6 (40.0)	11 (91.7)	23 (47.9)
2	4 (44.4)	2 (16.7)	5 (33.3)	1 (8.3)	12 (25.0)
3+	2 (22.2)	7 (58.3)	4 (26.7)	0	13 (27.1)

#### Professional practice factors

Dissatisfaction with the working environment was the reason most frequently cited by physicians of all generations for leaving a location. There were many elements which contributed to the quality of the work environment, including the collaborative nature of the practice, the relationship with administrators, and access to resources and personnel. In some cases, physicians described a poor work environment that stemmed from a lack of cooperation or collegiality among the physicians in a group practice:

“The physicians that I worked with all did their own thing and didn’t work together as a group. They didn’t take one another’s call, they all took their own call all the time, and in [community name] the economic situation wasn’t, the partnership arranged wasn’t appropriate.”

A poor work environment was also described in terms of the relationship with administrators or the amount of bureaucracy. A late-career physician, working in Manitoba, described this aspect of a poor work environment:

“You know I think the same thing happens in Saskatchewan as in Manitoba, I think most physicians are unhappy with resources, the hassle factor that you want to help people do the right thing but you are blocked, prevented, and obstructed when you try to do things, which is very frustrating… Hassles are different everywhere, but, there’s lots that one can do to makes things more pleasant, let’s say, which are not necessarily economic things.”

Likewise, a physician in Newfoundland described his frustration with the many layers and slow pace of administration:

“There’s a lot of politics here … there’s a lot of red tape and bureaucracy and stuff in trying to get stuff done. People are fairly slow to be progressive and that’s very frustrating whereas you don’t see that in bigger centres in the mainland so that’s definitely an issue.”

Lack of access to resources was also an important aspect of the work environment. Resources included operating room time, equipment, as well as other health care professionals. For example, in the following quotation, a mid-career physician describes the importance of updated equipment in the workplace:

*“*They’ve been pretty good with getting new equipment for us to work with here, and you can appreciate if you’re in radiology and you have 15 year old equipment that is not fun to work with, so updated equipment is always an important thing for maintaining job satisfaction.”

Similarly, physicians said that reducing turnover and having a sufficient staff to handle work load was important. For example, an early-career physician in Newfoundland noted the importance of having nursing staff to share the workload:

“I guess it comes down to providing the supports for whatever the person is being asked to do, and sometimes that means having additional people on staff with me who are able to help out, contribute to the callout schedule after hours, maybe a sub-specialist nurse who can help take phone calls from patients, that sort of thing could make a big difference.”

For many physicians, the work environment was identified as the key factor in their decision to remain or leave their current work location. A mid-career specialist suggested:

“I do think that [work environment] has a really high impact on physician as far as their working conditions, the collegiality, and actually who directs you, who really is your supervisor, who sets the standards.”

#### Economic factors

Two economic factors, remuneration and billing policies, were cited by some physicians as reasons for leaving a work location. Some physicians stated that income level was the key reason for leaving a location. For example, a Saskatchewan trained, late-career physician said of her decision to leave Quebec:

“… the health care system in Quebec then, and still now, is abysmal. They don’t pay physicians adequately, they don’t support hospitals or health care adequately in Quebec. So I thought, there’s no point in staying there, if I couldn’t afford to own my own house*.*”

For other physicians, income was the “last straw” in their decision to leave. For a female, mid-career, Memorial-trained specialist, in light of her unsatisfying work-life balance, her income convinced her to leave Newfoundland:

“But I’m also looking for something that gives me a better work/personal life balance. And currently it’s too much towards the work. And the remuneration is not there to make it worth while on that part. You know, if I was working really, really hard saying okay well I’m making lots of money doing it, so there is some trade off.”

While some physicians believed that remuneration in Saskatchewan and Newfoundland was less than other parts of Canada, others noted that the cost of living was also less. A surgeon working in Newfoundland offered the following observation:

“I am not a 100% sure I would do very much better than I do here. From the point of view of raising a family I don’t. I’ve never been anywhere else or worked anywhere else that I thought was exceedingly better than here.”

In the 1990s, Newfoundland introduced billing restrictions to encourage physicians to locate in rural areas. New physicians in urban areas were either not allowed to bill the provincial health insurance plan, or would only receive a proportion of the set fee [23]. Local billing policies influenced one late-career specialist to leave Newfoundland, despite his desire to remain in his home province.

“…the government had restrictions on billing numbers and I asked if there was any way around it. And there was no way that I could settle in St. John’s or Grand Falls or Gander or Corner Brook or any other large population center in Newfoundland in spite of the fact that I had graduated from Memorial and in spite of the fact that I had worked in rural Newfoundland particularly [community name] and in spite of my affiliation with the University.”

#### Other factors: political climate

Late and end-career physicians cited a political climate as an important influence in their decisions. Political climate refers to national or provincial policies as well as reaction to specific events. Some older generation physicians cited the local political situation for leaving a work location. For example a University of Saskatchewan trained, late-career physician cited the draft for leaving the United States: “…a draft lawyer advised me, if I left the country, before I was served with a draft notice, I wouldn’t be a draft dodger. And so that’s what we did… I was a sitting duck for the draft*”*. Another University of Saskatchewan trained, late-career physician spoke of the influence of the FLQ (Front de Libération du Quebec, a separatist group) crisis for her decision to leave Quebec: “I wanted to leave Montreal because of the political instability. It was during the time of the FLQ and separatism and all the rest of it. So I didn’t want to stay there*.*” An end-career physician also referred to the Medicare crisis and doctors’ strike for not working in Saskatchewan: “Plus of course this was not long after the Medicare crisis. So although I was willing to come back to Saskatchewan, there were a whole host of reasons, let’s put it this way, it didn’t take much to discourage me from going back to Saskatchewan.*”* Political climate was cited by men and women and by family physicians and specialists, but exclusively by end and late career physicians from Saskatchewan.

### Discussion

We used the framework proposed by Barer et al. [11] to guide the thematic coding of the interview transcripts. It is important to note that the framework was developed in order to organize and synthesize a large number of disparate, stand-alone studies that examined specific types of physicians or work places. We selected Barer et al.’s framework for this study because of its comprehensive nature and it’s applicability to the Canadian context. A number of other frameworks have been recently introduced that capture various policy interventions that have been used to improve the distribution of health care providers in rural and remote settings [24-26]. The factors in these newer frameworks, although they have names with different headings are similar to those in Barer et al.’s. Our future work will examine the relative importance of the factors in decisions to move to or leave a work location.

Few physician recruitment and retention studies use organizational behaviour theories to study turnover; the physician workforce has a number of unique characteristics that limit the applicability of these theories to it. For example, the majority of physicians in Canada work in privately owned, independent practices that are akin to small businesses [27]. The majority of physicians in Canada are not salaried [28] so many of the traditional aspects of the employee-employer relationship do not apply to most physicians.

One factor, political environment, did not fit within any existing categories in the framework [11]. Political environment refers to national or provincial policies or events, outside the health sector that affect the attractiveness of a region. In our study, some older-generation physicians reported that events (such as the FLQ crisis) or policies (such as the United States military draft) influenced where they chose to locate.

In our study, professional practice and economic factors influenced physicians’ decisions to leave a work location, regardless of generation, gender, specialty or medical school. A poor work environment was almost universally the most important reason why a physician chose to leave a location. As the quotations from participants illustrated, many elements contribute to the quality of the work environment. Many of these elements, such as inter-personal relationships between individual physicians or the management of privately owned practices, are largely outside the reach of public policy. This may explain in part why the work environment is rarely addressed in physician workforce planning documents [29-31]. Nonetheless, policies that address access to resources, staffing (of physicians and other health professionals), and administration may be effective means of improving physician retention, particularly at an institutional or regional level. Improving the work environment may be particularly important in provinces such as Saskatchewan and Newfoundland, where given their relatively small populations, there may be few organizations in the province where physicians may move if they are unsatisfied in their current workplace. For example, specialists may have no other choice but to leave the province to escape a poor workplace, whereas in more populous provinces, there may be a number of hospitals or regions where they may work. This finding may partially explain the high physician turnover in Saskatchewan and Newfoundland, particularly among specialist physicians.

The level of remuneration was identified by a few physicians as the reason they chose to leave a given province. However, it was more common for physicians to identify remuneration levels as inadequate when they were already unsatisfied with their work location, since physicians who are primarily motivated by economic factors were unlikely to settle in lower paying provinces in the first place. Although economic policies are often the focus of recruitment and retention initiatives [11], these findings suggest that economic polices alone may not address high turnover, particularly in the long-term. A recent review concluded that there were no high-quality studies that demonstrated that financial incentives were effective in improving the distribution of health workers in rural and remote areas [26].

#### Limitations

Recall bias is a limitation of any retrospective study relying on self reported data. In this study, participants may not have accurately remembered their reasons for choosing a location or deciding to leave. Their responses may also be influenced by social acceptability bias, that is, physicians may have felt obligated to give responses that are expected of them.

### Conclusion and future research

There are few generational differences in the reasons why physicians opt to leave a work location. The study draws attention to the importance of the work environment and organizational culture on the retention of physicians of all generations. While there may be limited public policies that can address the workplace environment of physicians in private practices, initiatives in other health care settings may contribute to improved retention. We also highlight the importance of political activism and climate to some older generation physicians. While economic factors have largely been the focus of recruitment and retention initiatives, our findings highlight the importance of multi-pronged strategies that address the range of factors identified in this study.

This study was part of a larger project examining the retention of physicians trained in Saskatchewan and Newfoundland. These qualitative interviews were followed by a survey of physicians trained in these provinces. In addition to providing an in-depth exploration of physician’s reasons for choosing and leaving a work location, the qualitative interviews also inform the next phase of the project. The surveys will be used to test the findings from this study on a larger, representative sample of physicians and assess the relative weight of the various factors in the decisions to move, remain in and/or leave a community. Together, these studies will provide a broader basis for policy development.

### Competing interests

The authors declare that they have no competing interests.

### Authors’ contributions

MM designed the study, analysed the data and prepared the drafts of the article. MS gathered data, helped analyse the data and provided feedback on the drafts of the article. NC gathered data and provided feedback on the drafts of the article. RTC provided feedback on the drafts of the article. All authors read and approved the final manuscript.
